# Laterality and sex differences in the expression of brain-derived neurotrophic factor in developing rat hippocampus

**DOI:** 10.1007/s11011-020-00620-4

**Published:** 2020-09-25

**Authors:** Reza Sardar, Zahra Zandieh, Zeinab Namjoo, Mansoureh Soleimani, Reza Shirazi, Javad Hami

**Affiliations:** 1 Department of Anatomy, Faculty of Medicine, Iran University of Medical Sciences, Tehran, Iran; 2 Cellular and Molecular Research Center, Faculty of Medicine, Iran University of Medical Sciences, Tehran, Iran; 3 Department of Anatomical Science, school of Medicine, Ardabil University of Medical Sciences, Ardabil, Iran; 4 Department of Health and Medical Sciences, Faculty of Health, Arts and Design, Swinburn University, Hawthorn, Melbourne Australia; 5 Cellular and Molecular Research Center, Birjand University of Medical Sciences, Birjand, Iran; 6 Institute for Anatomy and Cell Biology, Universitätsmedizin Greifswald, Greifswald, Germany

**Keywords:** Brain-derived neurotrophic factor, Lateralization, Hippocampus, Development

## Abstract

Brain-derived neurotrophic factor (BDNF), as a member of neurotrophin family, plays an important role in neurogenesis, neuronal survival and synaptic plasticity. BDNF is strongly expressed in the hippocampus, where has been associated with memory consolidation, learning, and cognition. In this study, Real-time PCR, immunohistochemistry, and stereology were used to evaluate the gender differences and left-right asymmetries in the expression of BDNF in the developing rat hippocampus during the neurogenesis-active period, at postnatal days P0, P7 and P14. We found the lowest expression of BDNF in the right side and the highest in the left side hippocampi of both male and female neonates at P14 (*P* ≤ 0.05 each). At the same time, there were significant differences in the hippocampal expression of BDNF between males and females (P ≤ 0.05 each). No important differences in the number of BDNF expressing neurons in different subregions of right/left hippocampus were observed between male and female animals at P0 and P7 (*P* > 0.05). Furthermore, the highest numerical density of BDNF positive cells was detected in the both sides hippocampal CA_1_ in the male/female offspring at P7, and in the CA_2_, CA_3_ and dentate gyrus at P14 (*P* ≤ 0.05 each). Based on these findings, it can be concluded that there are prominent sex and interhemispheric differences in the expression of BDNF in the developing rat hippocampus, suggesting a probable mechanism for the control of gender and laterality differences in development, structure, and function of the hippocampus.

## Introduction

Neuroanatomical and functional studies on central nervous system (CNS) have shown gender and interhemispheric asymmetries in some brain regions of both humans and rodents (Ocklenburg and Gunturkun 2012; Murphy et al. 1996; Ragbetli et al. 2002). These investigations have demonstrated the enormous complexity in the underlying molecular mechanisms involved in differentiation of left/right and male/female cerebral hemisphere, which is influenced by sexual hormones and genetic factors (Hou et al. 2013; Ocklenburg and Gunturkun 2012; Vogel et al. 2003; Gur and Gur 2017; Ruigrok et al. 2014). Hippocampus belongs to the limbic system and serves a critical function in long-term memory consolidation, learning, navigation and cognition. This region of the brain is composed of two major components, the hippocampus proper or Cornu Ammonis (CA regions) and dentate gyrus (DG). The CA area is divided into three subregions including CA_1_, CA_2_ and CA_3_. The various hippocampal areas differ in terms of efferents, afferents and major cell types, neurogenesis, and synaptic plasticity (Knowles 1992; Ormerod et al. 2007; Pawluski et al. 2009a; McBain 2008; Gilbert and Brushfield 2009). There is growing body of evidence supporting the existence of identifiable structural and functional laterality-and sex-differences in the hippocampus (Jäncke et al. 2015; Hou et al. 2013; Tabibnia et al. 1999; Ragbetli et al. 2002). Various lines of evidence indicate gender and lateralized differences in hippocampal volume and in the morphology of hippocampal pyramidal cells (Bayer 1980; Thompson et al. 2008; Murphy et al. 1996; Ragbetli et al. 2002). The striking variation in expression of 68 kinds of proteins between right and left hippocampi has already been exhibited in earlier studies (Hou et al. 2013). It has also reported that the right hippocampus in males is larger and thicker than the left side; In contrast to a thicker left hippocampus in the females’ brain (Diamond 1983; Tabibnia et al. 1999). Results of the study by Lister et al. (2006) have shown that the numbers of neurons in the right CA_1_ and CA_2/3_ areas of the rat brain are significantly less than of left hippocampus (Lister et al. 2006). Wimer and Wimer (1985) has also demonstrated a higher cell density of granule cell layer in the DG of male mice, suggesting a gender difference in the proliferation of hippocampal cells (Wimer and Wimer 1985). Nevertheless, the exact mechanism mediating the sexual and interhemispheric differences in structure and function of the hippocampus remains unclear (Bowers et al. 2010b; Pawluski et al. 2009a; Galea 2008; Ormerod et al. 2007).

Neurotrophins are a group of polypeptide growth factors which play critical roles in postnatal production, differentiation, and survival of neurons (Schinder and Poo 2000; Huang and Reichardt 2001; Greenberg et al. 2009; Leinninger et al. 2004; Bekinschtein and von Bohlen Und Halbach 2020). There are four major types of neurotrophins: Brain-derived Neurotrophic Factor (BDNF), Nerve Growth Factor (NGF), Neurotrophin-3(NT-3) and Neurotrophin-4/5 (NT-4/5) (Chourbaji et al. 2011; Bothwell 1995; Dhobale 2014). Among them, BDNF has been well studied for its important role in neuronal cell proliferation, differentiation, survival, synaptic connection and plasticity, particularly during development, and its important function in regulation of neuroplasticity, learning, memory, and cognition in adult brain (Eyileten et al. 2017; Bekinschtein et al. 2008; Flöck et al. 2016; Scharfman et al. 2005b; Ebuehi and Dibie 2015). Therefore, the availability of BDNF defines progenitor cell generation; increasing the amount of BDNF increases cell proliferation (Pencea et al. 2001) (Scharfman et al. 2005b), whereas reductions in the amount of BDNF decreases hippocampal neurogenesis (Lee et al. 2002). This action of BDNF is mediated through a low-affinity receptor “p75”. Mice that do not express p75 have fewer hippocampal stem cells, a smaller granule cell layer, and behavioral alterations (Young et al. 2007; von Bohlen Und Halbach and von Bohlen Und Halbach 2018; Bekinschtein and von Bohlen Und Halbach 2020).

In rodents, the highest levels of BDNF production and release have been verified in hippocampus (Eyileten et al. 2017; Meek et al. 2013; Murer et al. 2001; Tapia-Arancibia et al. 2004). Nevertheless, there is evidence demonstrating a variation in BDNF content in the hippocampus, ventromedial hypothalamus, cerebral cortex and amygdala between female and male rats. Male mice also show a higher expression of BDNF in the hippocampus, cerebral cortex and brain stem. Furthermore, there is no evidence demonstrating the sex differences in hippocampal expression of BDNF in human (Franklin and Perrot-Sinal 2006; Bland et al. 2005; Bakos et al. 2009; Szapacs et al. 2004; Hayley et al. 2015). Additionally, existing data suggest that molecular events which lead to sexual differentiation in the brain interact with BDNF pathways (Iwasa et al. 2016; Sun et al. 2005).

Regards to the essential role of the BDNF in regulation of developmental and cognitive functions in the hippocampus, we tested the hypotheses that the expression of BDNF may considered as a genetic factor that could causes gender and laterality differences in the hippocampus of rats. Hence, in the present study we compared the content of BDNF mRNA, as well as, the numerical density of BDNF reactive neurons in the both sides of developing male and female rat hippocampus at postnatal days P0, P7, and P14, using real-time PCR, immunohistochemistry staining and an unbiased stereological method. We chose these three time points since mounting evidence indicates the first two postnatal weeks corresponds to active period of neurogenesis, dendritic maturation, and synaptogenesis in the rodents’ brain.

## Materials and methods

### Animals

Female Wistar rats (200–250 g body weight) were purchased from Iran University of Medical Sciences (Tehran, Iran). Rats were housed in the controlled environment with light/dark cycle at 22 ± 1 °C for 1 week before beginning of experiment. All of the animal experiments were conducted according to international principal guidelines which were approved by local ethic committee for animal experiments at Iran University of Medical Sciences (IR.IUMS.FMD.REC1396.9511314003). Animals were mated with male adult Wistar rats overnight. After verification of pregnancy with observation vaginal plaque, male rats were separated from the pregnant females, and that day was considered as the first day of pregnancy. At the end of pregnancy, the animals were allowed to deliver naturally; the day of birth was defined as postnatal day 0 (P0). A total of 72 newborns from nine different litters were used in the present study. Pups were sexed and randomly assigned to three age groups (*n* = 24 each, twelve males and twelve females): P0, P7, and P14.

### Tissue processing

The pups were anesthetized with chloroform and killed by cervical dislocation at P0, P7, and P14. Their brains were rapidly removed, and then immersed in a fixative solution containing 4% paraformaldehyde and 0.1 M glutaraldehyde in 0.1 M phosphate buffer (pH 7.4) for 48 h at 4 °C. The brain tissues were processed by routine histological methods and embedded in paraffin blocks. Coronal sections (7-μm-thick) were cut using a sliding microtome (Leica, Buffalo Grove, IL). Sections including hippocampus from each animal were chosen and mounted on poly-L-lysine-coated slides for immunohistochemistry.

### Immunohistochemistry

Immunohistochemistry was performed for detection of BDNF in the rat neonates’ Hippocampi. In summary, hippocampus sections were deparaffinized with xylene, rehydrated through descending concentrations of ethanol, and rinsed in 0.1 M phosphate-buffered saline (PBS) for 10 min. The sections were then incubated with 1% hydrogen peroxide in PBS for 10 min at room temperature to inhibit endogenous peroxidase, washed three times with PBS, incubated for 1 h in 3% bovine serum albumin in Tris-buffered saline (TBS) of pH 7.4 at 22 °C, and incubated in primary rabbit polyclonal anti-BDNF (N20) (Santa Cruz Biotechnology, USA; Cat. No. sc-546) diluted 1:500 in TBS containing 1% bovine serum albumin for overnight at 4 °C. After washing, the brain sections were incubated with the secondary antibody, HRP-conjugated goat anti-rabbit IgG (1:500, Abcam, UK) for 2 h at room temperature. Control sections were stained without the primary antibody. After three washes in PBS and then in 50 mM Tris buffer (TB, pH 7.6), the sections were reacted with 0.05% diaminobenzidine tetrahydrochloride (DAB, Sigma-Aldrich, St. Louis, MO) solution in 50 mM TB containing 0.01% H2O2 for 5–15 min at room temperature. Finally, the sections were lightly counterstained with Mayer’s hematoxylin, dehydrated with graded concentrations of ethanol and xylene, and mounted.

### Stereological analysis

BDNF-immunoreactive cells within four distinct regions of the hippocampal formation (CA_1_, CA_2_, CA_3_, and DG) were counted by an investigator blinded to the protocol treatment, using the optical dissector technique described in detail by Sterio (1984) and Gundersen et al. (1988) (Sterio 1984; Gundersen et al. 1988). The optical dissector technique eliminates bias in counting as a result of cell size and shape. Briefly, the BDNF positive cells were counted as they came into focus while scanning through the section. For each section, eight to ten unbiased counting frames were sampled in a systematically random method inside the area of hippocampus including CA_1_, CA_2_, CA_3_, and DG. The preparations were examined under a light microscope using a X60 objective lens (Nikon, Germany) and images were transferred to computer using a high-resolution camera (BX51, Japan). The number of BDNF reactive neurons was counted using computer-generated counting frames. The mean numerical density of positive neuronal cells was estimated as follows:

$$ NA=\frac{\Sigma\ \mathrm{Q}}{\mathrm{a}/\mathrm{f}\cdotp \Sigma \mathrm{P}} $$

Where Σ-Q is the total number of counted BDNF-positive neurons in each section, a/f is the area of hippocampal region, and ΣP is the sum of frame-associated points hitting space.

### Quantitative reverse transcription PCR (qRT-PCR)

Neonates’ hippocampi were carefully dissected from the brain and preceded for molecular analysis. After homogenization, total RNA was extracted using the RNAx plus (SinaClon, Tehran, Iran), according to the manufacturer’s protocol, and resolved in 50 μl RNase-free water. RNA was analyzed by Nano-drop to define their concentration and purity. The denaturing gel electrophoresis method was used to test the RNA integrity. RNA was treated with DNase I (EN0521, Fermentas, Germany) to eliminate any DNA contamination. Purified RNA samples were converted into cDNA. cDNAs were synthesized with 1 μg of RNA, 0.5 μL of oligo dTs and 0.5 μL of random hexamer using cDNA Synthesis Kit (Prime Script RT Master Mix, TAKARA, Kyoto, Japan). All procedures were based on the manufacturer’s protocol. 1 μg of synthesized cDNA used for SYBR Green-based real-time RT-PCR via 2X qPCR kit (RR820L, Tli RNaseH Plus, TAKARA, Kyoto, Japan). Values from β-actin were used to loading normalization for each sample. Relative changes expression was determined using the (2^-ΔΔ*Ct*^) method relative to gene expression values for control rats. The primer sets were designed based on sequences from the NCBI database and checked for specificity using the NCBI BLAST tool (www.ncbi.nlm.nih.gov/BLAST); primers with no significant similarity to other loci were selected.

The following primers were used: BDNF: 5′-GGCTCTCATACCCACTAAGATACATC-3′ (forward) and 5′-CGGAAACAGAACGAACAGAAACAG-3′ (reverse) and β-actin 5′- CGGTCAGGTCATCACTATCGG-3′ (forward) and 5′- ATGCCACAGGATTCCATACCCA-3′ (reverse). Thermocycling parameters were: initial denaturation at 95 °C for 30 s, 40 cycles of 95 °C for 5 s and annealing and elongation at 60 °C for 30 s.

### Statistical analysis

Data were presented as the mean ± SD. To test the significance of the effects of postnatal day (P0 or P7 or P14), sex (male or female), and laterality (left or right) on the expression of BDNF mRNA, a three-way analysis of variance (ANOVA) was employed. A multivariate ANOVA was also conducted to examine the main and interaction effects of postnatal day, gender, laterality, and hippocampus subregion on the numerical density of BDNF-positive cells in immunohistochemistry method. Where a significant difference was detected (*P* ≤ 0.05), a Tukey’s post hoc test was performed. All statistical analyses were done using SPSS 22 software (SPSS Inc., Chicago, IL, USA).

## Results

### Numerical density of BDNF positive neurons in the hippocampal regions

**Fig. 1:**
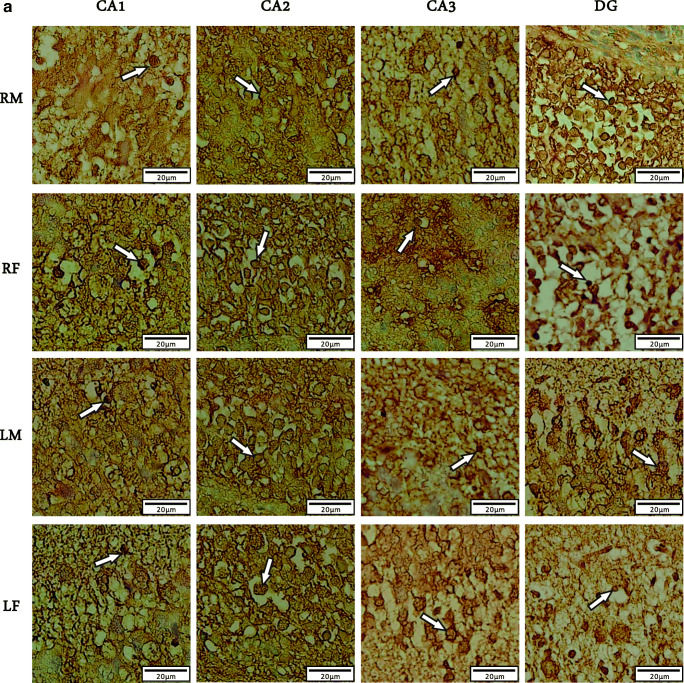

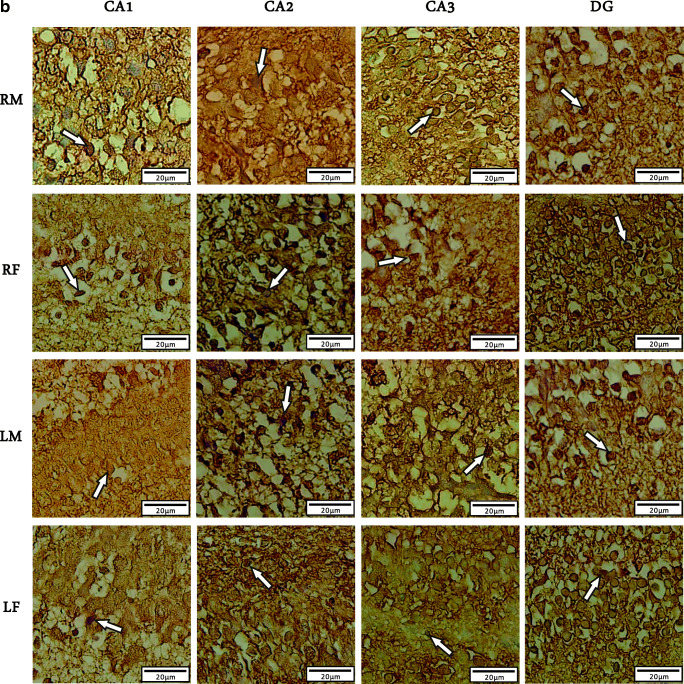

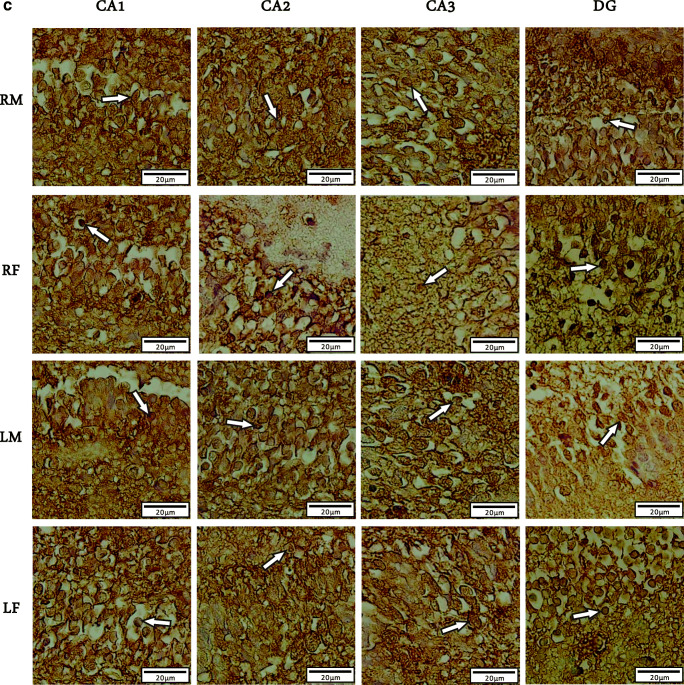
Photomicrographs showing distribution of BDNF-immunoreactive cells (white arrows) in different hippocampal areas of rat neonates on P0(**a**), P7(**b**), P14(**c**); (*n* = 10; 5 male and 5 female for each time point). *Abbreviations: RM, right male; RF, right female; LM, left male; LF, left female*

**Fig. 2:**
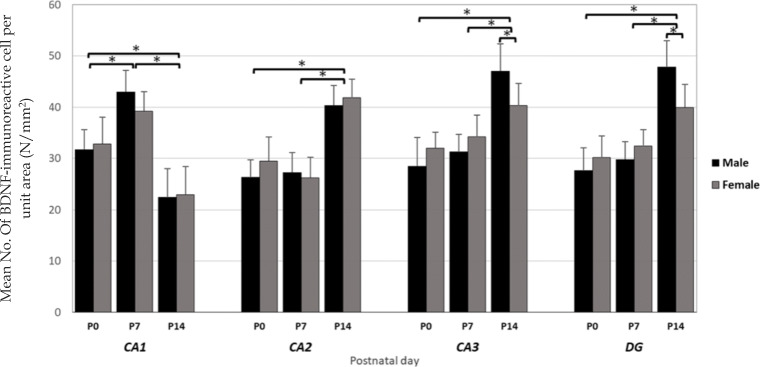
Numerical density of the BDNF positive cells in male and female hippocampal subareas CA_1_, CA_2_, CA_3_, and DG at postnatal days (P) 0, 7, and 14 in the right hippocampus. Values represent the mean ± SD. (n = 10; 5 male and 5 female for each time point). * *P* ≤ 0.05

**Fig. 3:**
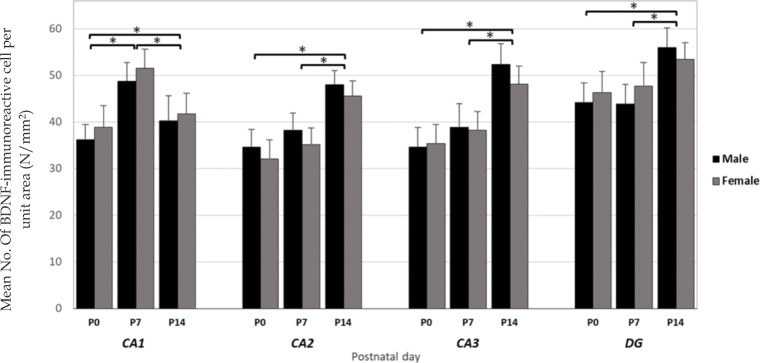
Numerical density of the BDNF positive cells in male and female hippocampal subareas CA_1_, CA_2_, CA_3_, and DG at postnatal days (P) 0, 7, and 14 in the left hippocampus. Values represent the mean ± SD. (n = 10; 5 male and 5 female for each time point). * P ≤ 0.05

**Fig. 4:**
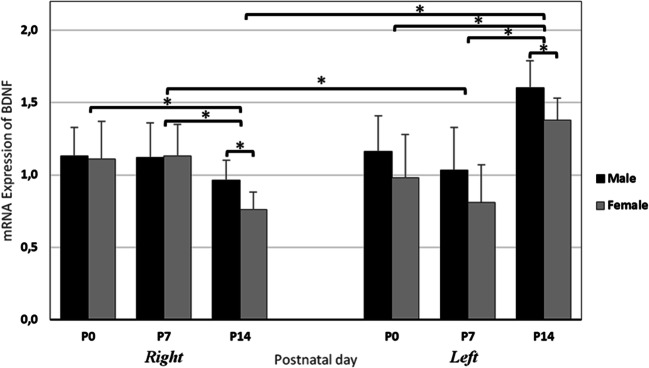
Differential expression of BDNF in right/left hippocampus of male/ female rat at postnatal days (P) 0, 7, and 14. Values represent the means ± SD. (*n* = 14; 7 male and 7 female for each time point). * P ≤ 0.05

We used Immunohistochemistry staining and stereology to determine the distribution pattern and numerical density of BDNF reactive neurons in various regions of right/left and male/female hippocampi of newborn rats (Fig. 1). A multivariate ANOVA was performed to investigate the effect of postnatal days (P0, P7 or P14), sex (male or female), laterality (left or right), and hippocampal subregions (CA_1_, CA_2_, CA_3_, or DG) on the mean number of BDNF-immunoreactive cells per unit area (N/mm^2^, Figs. 2 and 3). Summary of the results presented in Figs. 3 and 4. In spite of CA_1_ area, where the highest number of expressing cells was found in P7, the numerical density of BDNF-positive neurons was increased in the CA_2_, CA_3_, and DG at P14 in both sides’ hippocampi. Furthermore, there were no differences in the number of BDNF- reactive neurons between left and right hippocampus proper subregions at P0, whereas a higher number of immunoreactive cells observed in the DG of left hippocampi at the first day after birth. Our statistics revealed that there were significant differences in the number of BDNF-reactive cells related to postnatal day (F_2,34_ = 8.982, *p* = 0.003), laterality (F_1,34_ = 3.896, *p* = 0.012), and hippocampal area (F_1,34_ = 6.399, *p* = 0.001). Nevertheless, no significant effect of gender (F_1,34_ = 0.809, *P* = 0.075) was observed. A significant interaction was also detected between the postnatal day and laterality (F _3,21_ = 31.1; *p* = 0.01) but not between the hippocampal subarea and sex (F _2,23_ = 1.52; *p* = 0.33]. Post hoc analysis revealed a significant increase in the number of BDNF-positive neurons in all hippocampal areas of the left side in both male and female dams, when compared to right hippocampus at P7 (Male: CA_1_:*p* = 0.03; CA_2_:*p* = 0.014; CA_3_:p = 0.01; DG:*p* = 0.001; Female: CA_1_:*p* = 0.11; CA_2_:*p* = 0.02; CA_3_:p = 0.03; DG:p = 0.001) and P14 (Male: CA_1_:p = 0.001; CA_2_:p = 0.03; CA_3_:*p* = 0.003; DG:p = 0.001; Female: CA_1_:*p* = 0.04; CA_2_:*p* = 0.022; CA_3_:p = 0.001; DG:p = 0.001). Moreover, no significant differences were detected in the number of BDNF expressing neurons in different subareas of right hippocampus between male and female animals at P0 and P7 (*p* > 0.05 each). Our statistics also indicated a significantly lower number of BDNF expressing neurons in CA_3_ and DG areas of the right hippocampus in females at P14 (CA_3_:p = 0.02; DG:*p* = 0.003).

### Hippocampal BDNF mRNA expression

A quantification of mRNA using Real Time-PCR was carried out to assess the differential expression of hippocampal BDNF in various postnatal days (P0, P7, and P14), sex (male and female) and laterality (right and left), and data presented in Fig. 4. As noted, BDNF was highly expressed in the right hippocampus of male and female dams at P0 and P7, and its expression was decreased at postnatal day 14. In contrast, the level of BDNF mRNA expression in the left hippocampus of the male/female animals was upregulated at P14. The three-way ANOVA analysis showed a significant main effect of Laterality (F_1,19_ = 56.073, *p* = 0.018) and postnatal days (F_1, 19_ = 46.819, *p* = 0.003) but not sex (F_1, 19_ = 0.019, *p* = 0.8913) nor interaction (F1, 19) = 3.20, *p* = 0.08) on the expression of BDNF in the hippocampus of rat neonates. The *post-hoc* analysis indicated a significant lower expression of BDNF in both sides’ hippocampi of female animals compared with males in two weeks old animals (left: *p* = 0.011; right: *p* = 0.03). In addition, the expression of BDNF mRNA was markedly lower in left hippocampus, compared to right one at P7, regardless to the gender (*p* = 0.001).

## Discussion

To our knowledge, the current study is the first report investigating the expression of the BDNF, in developing hippocampus in order to determine the differential gene expression and region-specific distribution pattern between right-left and male-female utilizing real-time quantitative PCR, immunohistochemistry and stereological techniques. Based on substantial evidence demonstrating the pleiotropic effects of BDNF on neuronal development and synaptic plasticity that underlie hippocampal circuit formation and cognitive function (Bekinschtein et al. 2008; von Bohlen Und Halbach and von Bohlen Und Halbach 2018; Bekinschtein and von Bohlen Und Halbach 2020), the present study has investigated the possible effects of BDNF in the interhemispheric asymmetries and sexual variation of hippocampus in rat. Taken together, the results showed some gender- and laterality-differences in hippocampal expression of BDNF gene, and distribution pattern of expressing cells in various subareas during the first two postnatal weeks. This study was carried out at the first two postnatal weeks, when a very active hippocampal-dentate neurogenesis is occurred in rodents (Humphrey 1967). Brain development in newborn rats during this time period is similar to that seen in very preterm infants. In the hippocampus, the most prominent type of cells that determine the tri-synaptic circuitry are the pyramidal neurons that form the pyramidal layer of CA subregions, and the granule cells (GCs) of the DG. Earlier reports have illustrated the pyramidal neurons mainly formed before birth and GCs neurogenesis in the DG begins at embryonic day 15 (E15), and actively remains during the first postnatal weeks (Humphrey 1967; Altman and Das 1965; Schlessinger et al. 1975). Nevertheless, several lines of studies have shown a differential expression of some genes regulating the neurogenesis, differentiation, survival and synaptogenesis in the hippocampus of rodents during the first postnatal weeks (Hami et al. 2012; Hami et al. 2014a, b; Baradaran et al. 2020; Khoshdel-Sarkarizi et al. 2019). The studies by Hami and his colleagues (Hami et al. 2012, 2014a, b) have reported a significant interhemispheric asymmetry in expression and distribution of insulin-like growth factor-1 and insulin receptors (IGF-1R and InsR), as two important regulators of developmental and cognitive functions, in the developing rat hippocampus (Hami et al. 2014a, b, 2012). Regards to important roles of nicotinic acetylcholine receptors (nAChRs) subunits in development of nervous system; a recent study by Baradaran et al. (2020) has also investigated the lateralization of the α7 and α4 subunits of nAChRs in developing rat hippocampus. The authors have reported an increase in expression of both subunits from P0 to P7in the developing rat hippocampus. However, the expression of those subunits was decrease from P7 to P14 (Baradaran et al. 2020). Additionally, a series of important developmental events which exert influences on the hippocampal structure and function in rodents occurs at the first postnatal weeks (Stead et al. 2006; Moskal et al. 2006).

Our data obtained from real-time quantitative PCR indicated a differential pattern in expression of BDNF gene between either right and left or male and female developing hippocampi in rat. The highest level of BDNF mRNA expression was observed in the left side hippocampus of two weeks old rats, while the lowest expression was indicated in the right hippocampus of animals at the same age. In addition, the expression of BDNF mRNA was significantly lower in the both side hippocampi of female dams at P14 compared to males. Moreover, the mRNA expression of BDNF was markedly lower in left hippocampi than that of the right one in female animals at P7. Nevertheless, there were no significant laterality differences between male and female at P0 and P7. The results of the present investigation are consistent with the findings of prior study by Hami et al. (2012). In that study, the researchers have reported the highest value of IGF-1R expression at P7 in the right hippocampus and at P14 in the left one in male rats. In females, the peaked IGF-1R expression was occurred at P7 in both sides’ hippocampi. In contrast, they found an upregulation in hippocampal InsR expression in males at P14. Conversely, the expression of InsR in females peaked at P7 and then decreased again until P14 (Hami et al. 2012).

Similar to our results, there is a large body of evidence indicating that hippocampal pyramidal and dentate GCs express the BDNF (von Bohlen Und Halbach and von Bohlen Und Halbach 2018; Bekinschtein and von Bohlen Und Halbach 2020). Furthermore, our results have revealed a significant difference in numerical density of BDNF reactive neurons in left hippocampal CA_3_ and DG between male and female at two weeks age rats. In the right and left CA_2_, CA_3_, and DG areas of the male and female, the highest number of BDNF positive neuronal cells was observed at P14. Furthermore, the density of BDNF reactive cells of both sides’ hippocampal CA_1_ subregion in male and female was significantly higher on P7. An investigation by Hami et al. (2014)(Hami et al. 2014a) has reported a higher number of IGF-IR- positive cells in the left hippocampus of female at P7. By contrast, male pups have shown a significantly higher number of IGF-IR+ cells in the DG of the right hippocampus. The authors have also found an increase in the mean number of immunoreactive cells in CA_1_, CA_3_, and DG areas of left side hippocampus of males.

Early research on BDNF molecule found a relatively lower expression of BDNF during prenatal brain development in rodent, but then increases during the first few weeks after being born and peaks during the shift from embryonic to adult neurogenesis, especially in the hippocampus (Bath et al. 2012; Maisonpierre et al. 1990; Katoh-Semba et al. 1997). Maisonpierre and colleagues (Maisonpierre et al. 1990) characterized distribution of BDNF mRNA during rat development and found that the amount of it dramatically increased between embryonic days 11 and 12, with transcripts widely distributed by embryonic day 13. This provides key insight into its potential for facilitating neurogenesis, which then spurred much more research interest in its connection to neurogenesis. On the other hand, intraventricular BDNF application encourages neurogenesis in several parts of the rat brain(Pencea et al. 2001), and infusion directly into the hippocampus increases the number of granule cells in the DG (Scharfman et al. 2005a). Danzer et al. (2002) transfected hippocampal cells in culture with BDNF; GCs of DG exhibited considerable axonal and dendritic branching following BDNF. This effect was abolished with the application of a Trk receptor tyrosine kinase inhibitor, demonstrating that BDNF and Trk signaling promote neurogenesis both within and outside of the context of development. In line with this, a significant reduction in dendritic development, synaptic formation and maturation has been observed in postnatal-born granule neurons in different BDNF-mutant mice and BDNF secreted by newborn GCs has been shown to involve in dendrite development and synaptic maturation. TrkB-deficient mice have significantly fewer dendritic spines and excitatory synapses on CA_1_ neurons (Luikart et al. 2005; von Bohlen et al. 2008).

In order to determine the laterality differences in expression and regional distribution pattern of GABA receptors of developing rat hippocampus, Khoshdel-Sarkarizi et al. (2019) evaluated the postnatal expression of the two GABA_Aα1_ and GABA_B1_ subunits in the hippocampus of neonatal rats. Their results did not show a significant laterality differences in the general expression of these subunits, and there were only differences in distribution pattern of those receptor in hippocampal subregions (Khoshdel-Sarkarizi et al. 2019).

All results obtain from RT-PCR and immunohistochemistry in the present study were more or less following a similar pattern at the various studied time points. Nevertheless, there are evidence demonstrating no correlations between the number of some protein expressing neurons and their mRNA expression levels in the hippocampus of rodents in such studies. The authors have believed that this may happened due to axonal transport of proteins from their origin to target and shifting from one subtype to another during development (Zhang et al. 1998). This can explain why the differences in the gene expression of the receptors were minor at the protein level compared with the mRNA level in our study.

It is widely accepted that endogenous steroids mediate the gender difference in hippocampal cell proliferation (Pawluski et al. 2009b; Ormerod et al. 2004; Bowers et al. 2010a; Xiao and Jordan 2002), even though the exact mechanism remains unknown. Xiao and Jordan 2002) found the higher number of androgen receptor positive cells on the left than the right, and suggested that these variations may lead to laterality in hippocampal structure and function (Xiao and Jordan 2002). Our present results indicate the existence of a differential expression of BDNF in left–right and male–female developing rat hippocampus. Together with other mechanisms such as endogenous steroid levels, these differences may also underlie sexual dimorphism and left–right asymmetry in the hippocampus. Nevertheless, the hippocampus implicated in the etiology of several mental health disorders, many of which exhibit some degree of sex difference, and there are also subtle sex differences in hippocampal-associated behaviors such as spatial learning strategies and stress responsivity (Exner et al. 2008; Frazier et al. 2008; Kokkosis and Tsirka 2020; Krolick et al. 2018).

## Conclusions

In summary, our results indicate some sexual and interhemispheric differences in the mRNA expression and distribution pattern of the BDNF expressing neurons in the hippocampus of neonatal rats during the first two postnatal weeks. Although, the expression of the BDNF gene in the both sides’ hippocampi remain unchanged, regardless to the gender of animals, between P0 until P7; the expression and numerical density of BDNF positive neurons reached the maximum in left side hippocampal CA_2_, CA_3_, and DG subregions at P14. Given the crucial role of BDNF in the hippocampus development, these variations may be considered as a part of the cascades influencing the gender- and laterality differences in hippocampus structure and function.
