# Correction to: Laterality and sex differences in the expression of brain-derived neurotrophic factor in developing rat hippocampus

**DOI:** 10.1007/s11011-021-00779-4

**Published:** 2021-06-19

**Authors:** Reza Sardar, Zahra Zandieh, Zeinab Namjoo, Mansoureh Soleimani, Reza Shirazi, Javad Hami

**Affiliations:** 1 Department of Anatomy, Faculty of Medicine, Iran University of Medical Sciences, Tehran, Iran; 2 Cellular and Molecular Research Center, Faculty of Medicine, Iran University of Medical Sciences, Tehran, Iran; 3 Department of Anatomical Science, School of Medicine, Ardabil University of Medical Sciences, Ardabil, Iran; 4 Department of Anatomy, School of Medical Sciences, Medicine & Health, UNSW Sydney, Sydney, Australia; 5 Cellular and Molecular Research Center, Birjand University of Medical Sciences, Birjand, Iran; 6 Institute for Anatomy and Cell Biology, Universitätsmedizin Greifswald, Greifswald, Germany

## Abstract

A Correction to this paper has been published: https://doi.org/10.1007/s11011-021-00779-4

**Correction to: Metabollic Brain Disease (2021)**

**https://doi.org/10.1007/s11011-020-00620-4**

The article “Laterality and sex differences in the expression of brain-derived neurotrophic factor in developing rat hippocampus” written by Reza Sardar, Zahra Zandieh, Zeinab Namjoo, Mansoureh Soleimani,Reza Shirazi, Javad Hami, was originally published Online First without Open Access. After publication, the author decided to opt for Open Choice and to make the article an Open Access publication. Therefore, the copyright of the article has been changed to © The Author(s) 2021 and the article is forthwith distributed under the terms of the Creative Commons Attribution 4.0 International License (http://creativecommons.org/licenses/by/4.0/), which permits use, duplication, adaptation, distribution and reproduction in any medium or format, as long as you give appropriate credit to the original author(s) and the source, provide a link to the Creative Commons license, and indicate if changes were made.

The original article has been corrected.

Open Access This article is distributed under the terms of the Creative Commons Attribution 4.0 International License (http://creativecommons.org/licenses/by/4.0/), which permits unrestricted use, distribution, and reproduction in any medium, provided you give appropriate credit to the original author(s) and the source, provide a link to the Creative Commons license, and indicate if changes were made. The images or other third party material in this article are included in the article’s Creative Commons licence, unless indicated otherwise in a credit line to the material. If material is not included in the article’s Creative Commons licence and your intended use is not permitted by statutory regulation or exceeds the permitted use, you will need to obtain permission directly from the copyright holder. To view a copy of this licence, visit http:// creativecommons.org/licenses/by/4.0/.

